# Bone Mineral Density in Children and Adolescents of the Abay Region, Kazakhstan: Prevalence and Associated Risk Factors

**DOI:** 10.3390/ijerph22060949

**Published:** 2025-06-17

**Authors:** Madina Madiyeva, Gulnur Kanapiyanova, Gulzhan Bersimbekova, Mariya Prilutskaya, Alida Kaskabayeva, Tamara Rymbayeva, Altay Dyussupov

**Affiliations:** 1 Department of Radiology, Semey Medical University, Abay Street, 103, Abay Region, Semey 071400, Kazakhstan; madina.madiyeva@smu.edu.kz; 2 Department of Internal Diseases and Rheumatology, Semey Medical University, Abay Street, 103, Abay Region, Semey 071400, Kazakhstan; gulzhan.bersimbekova@smu.edu.kz (G.B.); alida.kaskabayeva@smu.edu.kz (A.K.); tamara.rymbayeva@smu.edu.kz (T.R.); 3 Department of Personalised Medicine, Pavlodar Branch of Semey Medical University, TorajgyrovStreet 72/1, Pavlodar Region, Pavlodar 140001, Kazakhstan; mariya.prilutskaya@smu.edu.kz; 4 Rector Office, Semey Medical University, Abay Street, 103, Abay Region, Semey 071400, Kazakhstan; altay.dyusupov@smu.edu.kz

**Keywords:** bone mineral density, children, adolescents, dual-energy X-ray absorptiometry, osteopenia, blood ionized calcium

## Abstract

Approximately 95% of skeletal size, bone, and muscle mass is achieved by the age of 18, with a rapid acceleration in bone mineral accumulation and muscle growth during the adolescent growth spurt. Bone mineral status in children and adolescents in Kazakhstan is a relevant subject for both clinical and fundamental research. The aim of this study was to investigate the prevalence of low bone mineral density (BMD) and the factors associated with it in children and adolescents living in the Abay region of Kazakhstan. The target group consisted of children and adolescents aged 5 to 17 years (*n* = 509) who had been residing in the Abay region of Kazakhstan since birth. Based on physiological age, participants were divided into two groups: 5–10 years (preschool and early school age) and 11–17 years (adolescence). All participants completed a questionnaire and underwent bone mineral density assessment using dual-energy X-ray absorptiometry (DXA). Comparisons were made between two age groups (5–10 and 11–17 years) and based on the presence or absence of reduced bone mineralization. Regression analysis identified four factors independently associated with reduced bone mineralization: ionized calcium (AOR 2099.9; *p* = 0.007), age (AOR 1.21; *p* = 0.013), body weight (AOR 0.97; *p* = 0.047), and green vegetables (AOR 0.46; *p* = 0.017). Conclusions: Our study demonstrated that 50.5% of children aged 5–10 years and 57.4% of adolescents aged 11–17 years had BMD below the age-specific norm. The study identified several risk factors that are associated with a decrease in BMD. These include blood ionized calcium, age, inadequate consumption of fruits and vegetables and dairy products, low physical activity, and insufficient sun exposure. These findings highlight the importance of early prevention of osteopenic conditions beginning in childhood and adolescence.

## 1. Introduction

Concerns regarding bone health are becoming an increasingly important issue for pediatricians [1]. Bone health depends on the continuous process of normal remodeling—a dynamic interaction in which bone resorption and formation harmoniously sustain microarchitecture, strength, and mineral balance. Any disruption to this balance can lead to dysregulated bone remodeling, potentially predisposing individuals to conditions involving bone loss, such as osteoporosis [2,3]. Although osteoporosis is typically considered a disorder of adulthood, it is becoming increasingly evident that its origins may trace back to childhood and adolescence [4]. Approximately 95% of skeletal size, bone, and muscle mass is achieved by the age of 18, with a rapid acceleration in bone mineral accumulation and muscle growth during the adolescent growth spurt [5,6]. The accumulation of bone minerals begins in the prenatal period and continues into the third decade of life—the developmental peak at which optimal bone mineralization is achieved. The intensive mineralization and growth of the skeleton is a significant biological phenomenon that plays a critical role in the development of peak bone mass [7]. Childhood and adolescence are critical periods for the attainment of optimal peak bone mass, which is considered the most effective strategy for preventing osteoporosis later in life [8,9]. By the end of puberty, bone mass in many skeletal regions approaches its maximum and determines the skeleton’s resistance to mechanical stress throughout life [10]. Due to the absence of official definitions, research over the past decades has not provided specific prevalence estimates of low bone mineral density (BMD) in various regions of the world, including Kazakhstan, among pediatric populations. In pediatric practice, the assessment of BMD—as in adults—depends on sex, race, ethnicity, and a range of population-specific risk factors [11]. In Kazakhstan, research on BMD remains limited, with only a single study conducted on a fixed age group of infants during their first year of life [12]. According to the International Society for Clinical Densitometry (ISCD), pediatric osteoporosis is currently defined by either of the following criteria: (1) a bone mineral density (BMD) Z-score ≤ −2 combined with a clinically significant fracture history, defined as two or more long bone fractures by age 10 or three or more long bone fractures at any age up to 10 years or (2) one or more vertebral compression fractures occurring in the absence of high-energy trauma or underlying local disease, regardless of the BMD Z-score [13]. Pediatric patients with genetic or acquired chronic conditions, as well as those with nutritional deficiencies, may fail to achieve expected gains in bone size, mass, and strength—placing them at increased risk of fractures [1]. While approximately 80% of bone mineral density is determined by genetic factors, the remaining 20% is influenced by modifiable elements such as nutrition, lifestyle, physical activity, body weight and composition, and hormonal status [14]. As such, adequate dietary intake of key minerals and vitamins—especially vitamin D and calcium—is essential, particularly during periods of rapid growth, such as childhood and adolescence. Vitamin D is a fat-soluble nutrient that plays a central role in calcium absorption and bone mineralization, showing a well-established positive correlation with BMD [15]. Deficiency in active vitamin D metabolites significantly impairs osteoid calcification, leading to rickets in children and adolescents or osteomalacia in adults [16]. Skeletal manifestations of vitamin D deficiency are also associated with a secondary factor—hyperparathyroidism—which results in increased bone remodeling, altered calcium and phosphate metabolism, and a heightened risk of osteopenia and osteoporosis [17]. Adequate intake of calcium and vitamin D is, therefore, essential for supporting optimal bone mass. Thus, the assessment of bone mineral status in children and adolescents is a subject of growing importance for both clinical and fundamental research. The aim of this study was to investigate the prevalence of low bone mineral density (BMD) and the factors associated with it in children and adolescents living in the Abay region of Kazakhstan.

## 2. Materials and Methods

### 2.1. Study Groups

All data for this study were collected between July 2023 and February 2025. The target group consisted of children and adolescents (*n* = 509) who were born and have been permanent residents of the Abay region of Kazakhstan. Participant recruitment was conducted at the clinical sites of the University Hospital of NJSC “Semey Medical University” and the “Toqtamys” Medical Center, located in the city of Semey—the administrative center of the Abay region. Parents or legal guardians of participating children and adolescents were informed about the nature and purpose of the study, and written informed consent was obtained prior to participation. Inclusion criteria were as follows: somatically healthy individuals aged 5 to 17 years whose sexual maturity corresponded to their biological age. Exclusion criteria were as follows: children under 5 years of age; children whose sexual maturity did not correspond to their biological age; any chronic diseases or congenital abnormalities of internal organs; recent bone trauma within the past three months; and individuals who declined participation in the study. Based on the WHO age classification (2025) and according to physiological development, participants were stratified into two age groups: 5–10 years (preschool and early school age) and 11–17 years (adolescents). This study was conducted in accordance with the principles of the Declaration of Helsinki and was approved by the Local Ethics Committee (LEC) of NJSC “Semey Medical University” (Protocol No. 7, dated 7 November 2022). This study was pre-registered in the international clinical trials registry on the website Clinicaltrials.gov, accessed on 27 March 2024 (ID NCT06344598).

### 2.2. Survey

Participant surveys were conducted offline and included personal ID, gender, age, place of residence, and the contact information of parents or legal guardians. The collected data were entered into a structured questionnaire. For all children and adolescents, height, body weight, and body mass index (BMI) were measured. Height was recorded with participants standing upright, attempting to touch the stadiometer with their head, back, buttocks, and heels. A single standardized device was used to measure body weight for all participants. The timing of puberty in children was assessed on the Tanner scale during a general medical examination by a pediatrician. The questionnaire included anamnestic information, such as breastfeeding history, history of bone fractures, hereditary musculoskeletal disorders, and fracture history in parents. Additional questions covered current use of vitamin D and calcium supplements, behavioral factors such as physical activity (regular sports participation), and daily outdoor exposure (defined as more than 20 min per day). Diet-related questions were categorized as “does not consume,” “consumes daily,” or “consumes irregularly.” The frequency of dairy product intake and consumption of nutrient-rich foods containing calcium, phosphorus, protein, and vitamin D was assessed based on age-appropriate daily requirements. For nutritional factor analysis, negative responses (“does not consume”) were emphasized. Survey reliability was evaluated by analyzing internal consistency, and content validity was assessed during the questionnaire development stage. A subset of respondents (*n* = 30) completed the questionnaire twice, and the percentage of response discrepancies was calculated.

### 2.3. DXA Measurement

Dual-energy X-ray absorptiometry (DXA) is the most widely used method for assessing bone mineral density (BMD). This technique is based on the quantitative measurement of X-ray absorption and the comparison of bone mineral content against age- and sex-matched reference standards. DXA delivers low-dose ionizing radiation and is a valuable tool for evaluating bone status, with well-established pediatric reference data [18,19]. In this study, BMD in children and adolescents was measured using an Osteosys DXA scanner (model 2020), focusing on the lumbar spine (L1–L4). Data were analyzed using the Z-score in accordance with the 2013 pediatric guidelines of the International Society for Clinical Densitometry (ISCD) [13]. The region of interest was selected either manually or automatically, depending on the clarity of anatomical landmarks. Areal BMD values (g/cm^2^) were converted into age, sex, and ethnic group in accordance with the available reference database of the DXA scanner on which the research was conducted with adjusted Z-scores. Interpretation of densitometric values followed the pediatric ISCD criteria in accordance with the available reference database of our Osteosys DXA scanner for the reference population: Z > 2 good; 2 > Z > 1 high; 1 > Z > 0 normal; 0 > Z > −1 low; −1 > Z > −2 lack; −2 > Z critical. For the purpose of this study, low bone mineral density was defined as lumbar spine BMD Z-score < −1.0 SD.

### 2.4. Biochemical Blood Analysis

To assess bone metabolism, serum levels of ionized calcium, vitamin D, and alkaline phosphatase were measured. Fasting venous blood samples were collected from each participant between 8:00 and 10:00 a.m., following an overnight fast. Laboratory analyses were conducted at the certified INVIVO laboratory (est. 2019). For vitamin D analysis, 5 mL of venous blood was collected in a vacuum tube with a clot activator. The level of 25-hydroxyvitamin D [25(OH)D] was determined using an immunochemiluminescence assay (ICMA) on the Beckman Coulter DXI800 analyzer (Indianapolis, IN, USA, 2020), with a reference range of 30–100 ng/mL. Values between 20 and 30 ng/mL were classified as insufficient, and levels below 20 ng/mL were considered deficient. For ionized calcium measurement, 5 mL of venous blood was collected in a vacuum tube with a clot activator and separation gel. Electrolyte levels were analyzed using a Roche AVL 9180 analyzer (Schaffhausen, Switzerland, 2019), with a reference range of 1.15–1.33 mmol/L. For serum alkaline phosphatase (ALP), another 5 mL blood sample was collected in a vacuum tube with a clot activator and gel. The concentration of ALP was measured using a colorimetric method on the Beckman Coulter AU5800 analyzer, with a reference range of 40–150 U/L. Sample processing followed standard laboratory protocols: clot formation occurred at room temperature for 30–45 min, followed by centrifugation at 2000–2200 rpm for 10 min. Serum samples were stored at +2 to +8 °C and analyzed within 72 h.

### 2.5. Statistical Data Processing

Descriptive and inferential statistical methods were employed. Categorical variables were summarized as absolute frequencies and percentages. Continuous variables were presented as mean ± standard deviation (SD) for normally distributed data or as median with interquartile range (IQR) when non-normality was observed. Group comparisons were conducted based on age (5–10 vs. 11–17 years) and the presence or absence of reduced bone mineralization. Categorical variables were compared using Pearson’s chi-square test. For continuous variables, the independent samples *t*-test was applied to normally distributed data, while the Mann–Whitney U test was used for data not meeting normality assumptions. To explore factors independently associated with reduced bone mineralization, binary logistic regression analysis was performed. The set of candidate predictors included demographic variables (age and sex), anthropometric characteristics (height, body weight, and body mass index), dietary behaviors, lifestyle factors (physical activity, sun exposure, and intake of calcium and vitamin D), and biochemical parameters (blood ionized calcium, 25-hydroxyvitamin D, and alkaline phosphatase). Given the relatively large number of predictors, the multivariate model was constructed using the backward conditional stepwise method, which sequentially removes non-significant variables based on likelihood-ratio criteria. This approach was selected to minimize model overfitting and to identify a parsimonious set of variables with independent explanatory value. Only covariates that demonstrated statistical significance were retained in the final adjusted model. Associations were reported as crude and adjusted odds ratios (ORs) with corresponding 95% confidence intervals (CIs). A two-tailed *p*-value < 0.05 was considered statistically significant. Effect sizes were calculated to quantify the magnitude of group differences. For continuous variables, Cohen’s d was used, and for categorical (binary) outcomes, odds ratios with 95% CIs were interpreted as effect size estimates. Effect size calculations were conducted using validated online statistical tools, including https://www.socscistatistics.com/effectsize/default3.aspx (accessed on 2 June 2025). All analyses were conducted using IBM SPSS Statistics, version 22 (IBM Corp., Armonk, NY, USA). https://www.psychometrica.de/effect_size.html (accessed on 2 June 2025).

## 3. Results

A total of 509 children and adolescents were examined. Of these, 258 were aged 5 to 10 years, and 251 were aged 11 to 17 years. The baseline characteristics of the participants in both groups are presented in Table 1. No statistically significant differences were found between the groups regarding behavioral factors. However, statistically significant differences were observed in age, body mass index (BMI), body weight, height, ionized calcium, and dietary patterns (dairy products) (*p* < 0.05).

**Figure 1:**
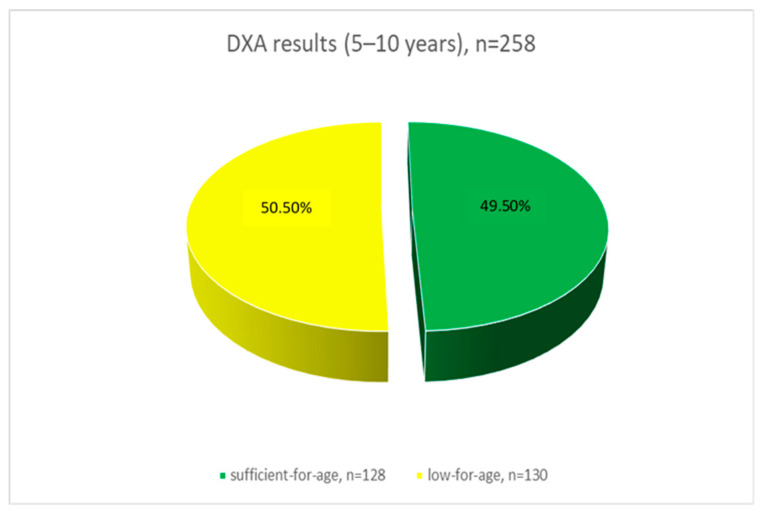
Densitometric indices in children aged 5–10 years (*p* < 0.05).

**Figure 2:**
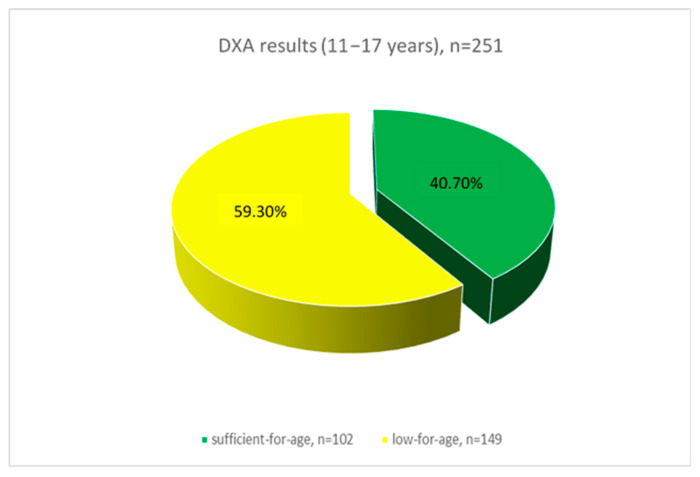
Densitometric indices in adolescents 11–17 years (*p* < 0.05).

According to the results of dual-energy X-ray absorptiometry (DXA), in the 5–10 years age group, 128 children (49.5%) had sufficient-for-age bone mineral density (BMD), while 130 children (50.5%) exhibited low-for-age BMD (Figure 1). In the 11–17 years age group, 102 adolescents (40.7%) demonstrated sufficient-for-age BMD, and 149 adolescents (59.3%) had low-for-age BMD (Figure 2).

In line with the objectives of our study, we formed a combined cohort of children aged 6 to 17 years, grouped according to normal and low bone mineral density. The general characteristics of risk factors in both groups are presented in Table 2. Comparative analysis between the two groups revealed statistically significant differences in age, height, ionized calcium, and nutrition (*p* < 0.05). Among children with normal BMD, 45.2% did not consume green vegetables, and 38.4% and 14% did not consume dried fruits and nuts, respectively (*p* < 0.05). The ionized calcium among all study participants remained within the normal reference range of 1.27 ± 0.07 mmol/L (*p* = 0.023). Furthermore, we found that both groups exhibited vitamin D deficiency, 16.19 ± 9.37 ng/mL. However, the difference between the groups was not statistically significant (*p* = 0.091).

Regression analysis of risk factors indicating a significant association with decreased bone mineral density (BMD) revealed several parameters affecting bone tissue mineralization. Among them, two parameters—age and blood ionized calcium—were associated with an increased likelihood of low bone density, while body weight and the consumption of green vegetables were associated with a decreased risk of bone tissue demineralization (Table 3).

## 4. Discussion

In our study, we examined somatically healthy children and adolescents divided into two age groups: 5–10 and 11–17 years. All participants underwent a bone mineral density test using dual-energy X-ray absorptiometry (DXA). The test compared two age groups (5–10 years and 11–17 years) based on the presence or absence of reduced bone mineralization.

Pediatric osteoporosis is rare and often secondary to a range of underlying conditions. Tan L.O. et al. [20] describe idiopathic juvenile osteoporosis as a primary form of the disease with unknown etiology that occurs in previously healthy children and is a diagnosis of exclusion, typically presenting with bone pain and fractures. The incidence is estimated at 1 per 100,000 children, with onset usually between the ages of 8 and 14 years—coinciding with the two to three years preceding puberty [20]. A significant increase in bone mass typically occurs by the third decade of life [21,22]. There are critical developmental windows during which heightened biological activity—alongside linear growth and bone tissue differentiation—leads to accelerated bone remodeling (resorption and formation). These periods, marked by rapid skeletal growth, occur during infancy, early childhood (ages 5–7), and puberty [2,3]. Although we did not identify statistically significant differences in body weight and height between the two age groups (*p* = 0.000), the rapid growth phases may represent a risk factor for reduced BMD [3].

It is well established that bone health is influenced by multiple factors, including sex, ethnicity, dietary intake of calcium, vitamin D, sodium, and protein, total body fat, hormonal status, and level of physical activity. BMI also affects the health of bone: individuals with higher body mass experience greater mechanical loading on the skeleton, which may result in stronger bones. Van Leeuwen et al. (2017) reported significantly higher BMD in overweight and obese children compared to those of normal weight [23]. However, other studies have shown that excess weight in children is associated with elevated levels of leptin and parathyroid hormone (PTH), both of which may enhance bone resorption and increase the risk of osteoporosis and fractures [24]. Our statistical analysis revealed that BMI was a significant factor across the age groups (*p* = 0.000). However, the mean BMI (17.8 ± 3.9 kg/m^2^) in both age groups remained within normal limits, indicating that neither overweight nor underweight status was prevalent in our study population.

The role of nutrition in bone health has been highlighted by numerous authors [12,25,26,27]. In general, the consumption of fruits and vegetables has a protective effect on overall health, particularly through the prevention of inflammation and associated conditions, including bone loss. These protective effects are primarily attributed to the bioactive components present in these foods. According to Villareal et al. (2015), diets high in potassium-rich foods—such as fruits and vegetables—are associated with a lower dietary acid load, which correlates with reduced bone resorption and thus supports skeletal health [28]. In our study, based on questionnaire data from 509 children and adolescents, 223 (43.8%) reported not consuming vegetables, while only 5.3% and 7.9% stated they did not consume meat and milk daily. These findings highlight the urgent need to revise dietary habits among children and adolescents, promoting increased intake of fruits and vegetables.

Another common cause of osteopenia in children and adolescents is physical inactivity. According to the mechanostat theory, bone strength is regulated by muscle force. During periods of immobility, the absence of muscle tension leads to decreased biomechanical loading of bones. This reduction is sensed by osteocytes and translated into biochemical signals that result in the thinning of long bones and reduced formation of trabecular bone [29]. Although athletic training is essential for healthy bone development, not all sports positively affect skeletal mass. Sports activities can be broadly categorized as osteogenic (weight-bearing exercises) and non-osteogenic (non-weight-bearing exercises). Among its many health benefits, football is considered an osteogenic sport and contributes to increased bone mass in both children and adolescents. In contrast, activities such as swimming or cycling do not significantly affect—or may even reduce—bone mass, potentially hindering the attainment of peak bone mass and compromising long-term bone health [20]. In a study by Sayar Y. et al. [29], the impact of physical activity on BMD in adolescents aged 9 to 18 years was investigated. Participants were divided into two groups based on levels of regular physical activity. Serum levels of calcium (Ca^2+^), phosphorus (P), vitamin D, and alkaline phosphatase (ALP) were measured. The study found that vitamin D levels were higher in the physically active group. Although no statistically significant difference in the prevalence of osteopenia (Z-score < −1) was observed between groups, physical activity appeared to enhance BMD without significantly lowering it [29]. Other authors have concluded that regular physical activity, when combined with high calcium intake, is beneficial for bone health in individuals aged 3 to 18 years [30]. In our study, 290 out of 509 children (57%) reported not attending any sports clubs or training sessions—an alarming trend that may negatively impact bone health in Kazakhstani youth.

Sunlight exposure to the skin of the hands, limbs, and face for at least 6–8 min per day in summer and approximately 30 min per day in autumn and winter is considered critical for endogenous vitamin D synthesis [31]. Our questionnaire revealed that 38 children (7.5%) do not receive daily sun exposure. The mean serum vitamin D level was 14.87 ng/mL among adolescents and 17.01 ng/mL among children, indicating a heightened risk of developing osteopenia in the future. Since the primary sources of vitamin D are sunlight and diet—and only a limited number of foods contain adequate amounts—insufficient sun exposure presents a significant health concern [25]. A study conducted in the western region of Kazakhstan in 2019 also investigated the impact of UV radiation and outdoor activity on vitamin D levels. The authors identified widespread vitamin D deficiency among children and concluded that this condition is most prevalent in temperate regions with limited sunlight exposure [32]. The eastern region of Kazakhstan, where our study was conducted, similarly experiences prolonged periods of reduced solar radiation (http://www.pogodaiklimat.ru/climate/36177.htm) (accessed 1 May 2025), which likely contributes to the observed vitamin D deficiency. Vitamin D deficiency impairs calcium absorption and leads to elevated levels of parathyroid hormone (PTH), which in turn triggers calcium resorption from the bone matrix, ultimately resulting in decreased bone mass. According to the results of the DXA, 50.5% of children aged 5–10 have low bone mineral density (BMD) for their age group. In the 11–17 year age group, 57.4% of adolescents have low-for-age BMD. In our study, children with low bone mineral density had vitamin D deficiency in blood serum—15.32 (ng/mL). Children with sufficient-for-age bone mineral density had insufficiency of vitamin D (16.87 (ng/mL)), but no statistical differences were found between vitamin D levels and bone density. Similarly, no differences were found between alkaline phosphatase (ALP) and BMD.

It is well established that chronic vitamin D deficiency during childhood and adolescence increases the risk of early-onset osteoporosis [33]. According to our data, 72.7% of participants did not report taking vitamin D supplements, which may partly explain the overall low serum vitamin D levels in this population. Interestingly, the blood ionized calcium concentrations remained within the reference ranges for the entire cohort. Analysis of the ionized calcium levels in two groups, one with sufficient BMD and the other with reduced BMD, revealed a statistically significant difference (*p* = 0.023).

In our final multivariate logistic regression model, blood ionized calcium was associated with an increased likelihood of low BMD.

## 5. Limitation

This study was a screening-based investigation and, as such, has several limitations. Data collection was conducted during a specific time period, and participants were grouped solely by chronological age without consideration of pubertal stages (Tanner stages). The next limitation was a lack of reference data that appropriately reflect the ethnic and age-specific characteristics of the pediatric population in Kazakhstan. Nevertheless, our results can still be useful for estimating converted values.

Additionally, a notable limitation of our study was the refusal of parents to permit blood sampling for biochemical analysis in preschool and early school-age children with “sufficient for age” BMD. As a result, regression analyses involving biochemical variables were conducted on a subsample of participants with complete data (*n* = 186). Cases with missing biochemical data were excluded using listwise deletion, which is a standard approach in regression modeling when key predictor or outcome variables are missing. Given that these missing data were primarily due to logistical constraints rather than systematic differences between participants and that imputation of complex biochemical markers could introduce bias, we opted for a complete-case analysis. The causal relationships identified in our study are tentative and require confirmation through cohort study designs.

Furthermore, the sample consisted of a limited number of children and adolescents from a single geographic region (Abay Region, Kazakhstan), which may restrict the generalizability of the findings to other regions or populations.

## 6. Conclusions

Our study demonstrated that 50.5% of children aged 5–10 years and 57.4% of adolescents aged 11–17 years had bone mineral density (BMD) below the age-specific norm. We associate the more pronounced changes in the older age group with the pubertal period.

Regression analysis of risk factors significantly associated with reduced BMD identified several parameters influencing bone mineralization. Among these, age ([AOR] = 1.21; *p* = 0.013) and blood ionized calcium ([AOR] = 2009.9; *p* = 0.007) were associated with an increased likelihood of low BMD, whereas body weight ([AOR] = 0.97; *p* = 0.047) and regular consumption of green vegetables ([AOR] = 0.46; *p* = 0.017) were associated with a reduced risk of bone demineralization.

The study also revealed several unfavorable lifestyle factors among the examined children and adolescents, including insufficient intake of fruits and vegetables, low consumption of dairy products, low levels of physical activity, and inadequate exposure to sunlight.

These findings highlight the importance of early prevention of osteopenic conditions beginning in childhood and adolescence. Preventive strategies should aim to modify risk factors such as diet, physical activity, and adequate sun exposure.

## Figures and Tables

**Table 1:** The characteristics of participants and comparison across age groups.

Parameter	Total (*n* = 509)	5–10 Years (*n* = 258)	11–17 Years (*n* = 251)	Statistical Criterion		Effect Size
				*t*-Test	*p*-Value	Cohen’s d
Age (Years)	10.7 ± 3.4	7.8 ± 1.6	13.6 ± 1.9	37.3	0.000	3.31
BMI (kg/m^2^)	17.8 ± 3.9	16.2 ± 2.9	19.4 ± 4.3	9.8	0.000	0.87
Weight (kg)	36.9 ± 15.5	26.14 ± 7.47	47.91 ± 13.79	22.1	0.000	1.96
Height (cm)	141.1 ± 19.8	126.35 ± 12.27	156.35 ± 13.61	26.1	0.000	2.31
	*n* (%)	*n* (%)	*n* (%)	χ^2^		OR (95% CI)
Male	268 (52.7%)	132 (51.2%)	136 (54.2%)	0.466	0.495	0.89 (0.63; 1.26)
Female	241 (47.3%)	126 (48.8%)	115 (45.8%)			
Not breastfed	35 (6.9%)	17 (6.6%)	18 (7.2%)	0.067	0.795	0.91 (0.46; 1.82)
* Fractures	81 (15.9)	34 (13.2%)	47 (18.7%)	2.925	0.087	0.66 (0.41; 1.07)
** Heredity	25 (4.9%)	14 (5.4%)	11 (4.4%)	0.297	0.586	1.25 (0.56; 2.81)
Parents’ fractures	96 (18.9%)	41 (15.9%)	55 (21.9%)	3.014	0.083	0.67 (0.43; 1.05)
Behavioral factors						
^1^ Vitamin D	370 (72.7%)	185 (71.7%)	185 (73.7%)	0.256	0.613	0.91 (0.62; 1.34)
^1^ Calcium suppl.	410 (80.6%)	206 (79.8%)	204 (81.3%)	0.166	0.684	0.91 (0.58;1.41)
^2^ Sport sections	290 (57.0)	149 (57.8%)	141 (51,66.2)	0.129	0.719	1.07 (0.75; 151)
^3^ Daily walks	38 (7.5%)	17 (6.6%)	21 (8.4%)	0.563	0.453	0.78 (0.40; 1.51)
Nutritional intake						
^4^ Milk and dairy products	40 (7.9%)	14 (5.4%)	26 (10.4%)	4.27	0.04	0.49 (0.25; 0.98)
^4^ Green vegetables	223 (43.8%)	119 (46.1%)	104 (41.4)	1.14	0.29	1.21 (0.85; 1.72)
^4^ Meat	27 (5.3%)	15 (5.8%)	12 (4.8%)	0.28	0.60	1.23 (0.56; 2.69)
^4^ Fish and seafood	98 (19.3%)	49 (19.1%)	49 (19.5%)	0.01	0.91	0.98 (0.63; 1.52)
^4^ Dried fruits and nuts	88 (17.3%)	47 (18.3%)	41 (16.3%)	0.34	0.56	1.15 (0.72; 1.82)
^4^ Eggs	59 (11.6%)	31 (12%)	28 (11.2%)	0.09	0.76	1.08 (0.63; 1.87)
^5^ Soda	117 (23.0%)	61 (23.7%)	56 (22.3%)	0.15	0.70	1.08 (0.72; 1.64)
^5^ Fast food	56 (11.0%)	26 (10.1%)	30 (12.0%)	0.44	0.51	0.83 (0.48; 1.45)
Blood analysis	(*n* = 186)	(*n* = 82)	(*n* = 104)	U	*p*	Cohen’s d
Vitamin D, ng/mL	16.19 ± 9.37	17.01 ± 10.53	14.87 ± 7.35	3271.5	0.006	0.41
Alkaline phosphatase, U/L	226.56 ± 135.38	239.93 ± 87.83	182.17 ± 172.67	3310.5	0.009	0.39
Ionized calcium, mmol/L	1.27 ± 0.07	1.28 ± 0.05	1.26 ± 0.08	3244.5	0.005	0.42

* Fracture after minor injury or a fall (2 or more before 10 years or 3 before 19 years). ** Hereditary disease of the musculoskeletal system in one of the parents. ^1^ Not taking at the moment; ^2^ not participating apart from school; ^3^ does not happen in the fresh air for more than 20 min a day; ^4^ does not consume; ^5^ consumes daily.

**Table 2:** Group characteristics by bone mineral density.

Parameter	Total	Sufficient for Age Z ≥ −1.0	Low for Age Z < −1.0	Statistical Criterion		Effect Size
	(*n* = 509)	(*n* = 230)	(*n* = 279)	*t*-Test	*p*-Value	Cohen’s d
Age (Years)	10.7 ± 3.4	10.21 ± 3.29	11.04 ± 3.48	2.74	0.006	0.25
Weight (kg)	36.9 ± 15.5	35.58 ± 15.16	37.95 ± 15.74	1.72	0.09	0.15
Height (cm)	141.1 ± 19.8	139.18 ± 19.33	142.76 ± 20.1	2.04	0.04	0.18
BMI (kg/m^2^)	17.8 ± 3.9	17.62 ± 4.03	17.91 ± 3.97	0.84	0.41	0.07
	*n* (%)	*n* (%)	*n* (%)	χ^2^		OR (95% CI)
Male	268 (52.7%)	127 (55.2%)	141 (50.5%)	1.108	0.293	1.21 (0.85; 1.71)
Female	241 (47.3%)	103 (44.8%)	138 (49.5%)			
Not breastfed	35 (6.9%)	21 (9.1%)	14 (5.0%)	3.330	0.068	1.90 (0.94; 3.83)
* Fractures	81 (15.9%)	32 (13.9%)	49 (17.6%)	1.255	0.263	0.75 (0.47; 1.23)
** Heredity	25 (4.9%)	12 (5.2%)	13 (4.7%)	0.084	0.772	1.12 (0.50; 2.52)
Parents’ fractures	96 (18.9%)	42 (18.3%)	54 (19.4%)	0.099	0.75	0.93 (0.59; 1.46)
Behavioral factors						
^1^ Vitamin D	370 (72.7%)	170 (73.9%)	200 (71.7%)	0.315	0.574	0.89 (0.60; 1.32)
^1^ Calcium suppl.	410 (80.6%)	189 (82.2%)	221 (79.2%)	0.706	0.401	0.82 (0.53; 1.29)
^2^ Sport sections	290 (57.0%)	126 (54.8%)	164 (58.8%)	0.822	0.364	0.85 (0.60; 1.21)
^3^ Daily walks	38 (7.5%)	17 (7.4%)	21 (7.6%)	0.005	0.945	0.98 (0.50; 1.90)
Nutritional intake						
^4^ Milk and dairy products	40 (7.9%)	16 (7.0%)	24 (8.6%)	0.471	0.492	0.79 (0.41; 1.53)
^4^ Green vegetables	223 (43.8%)	116 (50.4%)	107 (38.4%)	7.48	0.006	1.64 (1.14; 2.33)
^4^ Meat	27 (5.3%)	12 (5.2%)	15 (5.4%)	0.008	0.929	0.97 (0.44; 2.11)
^4^ Fish and seafood	98 (19.3%)	49 (21.3%)	49 (17.7%)	1.053	0.305	1.25 (0.81; 1.96)
^4^ Dried fruits and nuts	88 (17.3%)	49 (21.3%)	39 (14.0%)	4.652	0.031	1.93 (1.22; 3.05)
^4^ Eggs	59 (11.6%)	27 (11.7%)	32 (11.5%)	0.009	0.925	1.02 (0.60; 1.77)
^5^ Soda	117 (23%)	51 (22.2%)	66 (23.7%)	0.17	0.676	0.91 (0.60; 1.39)
^5^ Fast food	56 (11.0%)	26 (11.3%)	30 (10.8%)	0.034	0.854	1.05 (0.60; 1.84)
Blood analysis	(*n* = 186)	(*n* = 70)	(*n* = 116)	U	*p*	Cohen’s d
Vitamin D, ng/mL	16.19 ± 9.37	16.87 ± 8.47	15.32 ± 10.28	3458.0	0.091	0.25
Alkaline phosphatase, U/L	226.56 ± 135.38	235.93 ± 123.64	225.82 ± 154.93	3873.0	0.599	0.08
Ionized calcium, mmol/L	1.27 ± 0.07	1.26 ± 0.08	1.27 ± 0.08	3251.5	0.023	0.34

* Fracture after minor injury or a fall (2 or more before 10 years or 3 before 19 years). ** Hereditary disease of the musculoskeletal system in one of the parents. ^1^ Not taking at the moment; ^2^ not participating apart from school; ^3^ does not happen in the fresh air for more than 20 min a day; ^4^ does not consume; ^5^ consumes daily.

**Table 3:** Regression analysis of risk factors indicating significant association with decreased BMD (*n* = 509).

Parameter	OR	95% CI	*p*	AOR	95% CI	*p*
Age	1.07	1.02; 1.13	0.007	1.21	1.05; 1.41	0.013
Weight (kg)	1.01	0.99; 1.022	0.087	0.97	0.94; 1.00	0.047
Height (cm)	1.009	1.00; 1.02	0.043	-	-	-
BMI (kg/m^2^)	1.02	0.98; 1.07	0.404	-	-	-
Sex, male	0.83	0.58; 1.18	0.29	-	-	-
^1^ Vitamin D	0.89	0.61; 1.32	0.574	-	-	-
^1^ Calcium suppl.	0.83	0.53; 1.29	0.401	-	-	-
^2^ Sport sections	1.18	0.83; 1.68	0.37	-	-	-
^3^ Daily walks	1.02	0.53; 1.99	0.945	-	-	-
^4^ Milk and dairy products	1.26	0.65; 2.43	0.493	-	-	-
^4^ Green vegetables	0.61	0.43; 0.87	0.006	0.46	0.24; 0.87	0.017
^4^ Meat	1.04	0.48; 2.26	0.929	-	-	-
^4^ Fish and seafood	0.79	0.51; 1.23	0.305	-	-	-
^4^ Dried fruits/nuts	0.60	0.38; 0.96	0.032	-	-	-
^4^ Eggs	0.97	0.57; 1.68	0.925	-	-	-
^5^ Soda	1.09	0.72; 1.66	0.676	-	-	-
^5^ Fast food	0.95	0.54; 1.66	0.854	-	-	-
Blood analysis (*n* = 186)						
Alkaline phosphatase, U/L	1.001	0.998; 1.004	0.374	-	-	-
Vitamin D, ng/mL	0.98	0.95; 1.003	0.084	-	-	-
Ionized calcium, mmol/L	632.5	4.09; 97,767.8	0.012	2099.9	7.77; 567,476	0.007

^1^ Not taking at the moment; ^2^ not participating apart from school; ^3^ does not happen in the fresh air for more than 20 min a day; ^4^ does not consume; ^5^ consumes daily.

## Data Availability

The data necessary to reproduce the results presented here are not publicly accessible as the participants’ informed consent did not include public data sharing but are available from the first author upon reasonable request.
